# Professional perspectives on roles and structural gaps in interprofessional collaboration for suicide prevention: a qualitative study

**DOI:** 10.3389/fpsyt.2026.1724853

**Published:** 2026-03-09

**Authors:** Eva Hollenstein, Dolores Angela Castelli Dransart, Sarah Rajkumar, Michael Durrer, Anja Gysin Maillart, Laurent Michaud, Stéphane Saillant, Kaspar Wyss

**Affiliations:** 1 Swiss Centre for International Health, Swiss Tropical and Public Health Institute, Allschwil, Switzerland; 2 Faculty of Medicine, University of Basel, Basel, Switzerland; 3 School of Social Work Fribourg, HES-SO University of Applied Sciences and Arts Western Switzerland, Fribourg, Switzerland; 4 Lucerne Psychiatry, Lucerne, Switzerland; 5 Translational Research Centre, University Hospital of Psychiatry, University of Bern, Bern, Switzerland; 6 Unit for Clinical Suicide Research, Department of Clinical Sciences, Psychiatry, Faculty of Medicine, University of Lund, Lund, Sweden; 7 Department of Medical Psychology and Medical Sociology, University of Leipzig, Leipzig, Germany; 8 Psychiatric Liaison Service, Lausanne University Hospital (CHUV) and University of Lausanne, Lausanne, Switzerland; 9 Faculty of Biology and Medicine, University of Lausanne, Lausanne, Switzerland; 10 Emergency-Liaison-Hospital Service, Department of Adult Psychiatry, Neuchâtel Psychiatry Centre, Neuchâtel, Switzerland

**Keywords:** care coordination, interprofessional collaboration, mental health services, professional perspectives, qualitative research, suicide prevention, Switzerland

## Abstract

**Introduction:**

Suicidal behavior results from a complex interplay of psychiatric, psychosocial, socioeconomic, and structural factors and requires coordinated approaches across care sectors. Interprofessional collaboration (IPC) is widely regarded as essential for bridging gaps in care, yet profession-level descriptions of how IPC is organized and where structural bottlenecks occur in suicide prevention remain limited. This study examined how IPC is structured and experienced from the perspective of mental health professionals involved in selected suicide prevention programs in Switzerland.

**Methods:**

Semi-structured interviews were conducted with 15 professionals, including psychiatrists, psychologists, psychotherapists, and psychiatric nurses. Interviews were audio-recorded, transcribed verbatim, pseudonymized, and analyzed using thematic analysis. A complementary perception-based network visualization depicted reported collaboration patterns.

**Results:**

Participants described a layered collaboration structure with a clinical core comprising psychiatrists, psychologists, and psychiatric nurses, who assumed central responsibility for crisis assessment, therapeutic interventions, and follow-up care. General practitioners and social workers acted as bridging actors at transition points, while collaboration with actors such as teachers, police officers, and probation officers was described as more uncertain and episodic. IPC was inconsistently organized: some services relied on designated key workers, whereas others managed collaboration *ad hoc*. Task redistribution, shared training, and information-sharing facilitated collaboration, while limited resources, fragmented documentation systems, and the absence of clearly defined coordination roles remained major barriers.

**Discussion:**

From a professional perspective, IPC in Swiss suicide prevention is anchored in specialist mental health services but depends on wider cross-sectoral collaboration. Strengthening IPC requires formal coordination roles, interoperable documentation, and durable structures that embed collaboration across care sectors.

## 1 Introduction

Suicidal behavior results from a complex interplay of psychiatric, psychosocial, socioeconomic, environmental, and structural factors, including the availability of means, cultural norms, systemic inequalities, and barriers to care, requiring coordinated responses from diverse health and social care providers (1). Therefore, suicide prevention depends on the collaboration of professionals from different fields – such as mental health, primary care, and social services – working across various levels of mental health service provision.

Many individuals who die by suicide have had recent contact with health providers, particularly within psychiatric services or primary care (2, 3). Periods of high suicide risk occur at care transitions, where even short-term disruptions in coordination, communication, or follow-up can have serious consequences. A well-documented example is following discharge from psychiatric inpatient units: At this transition, the epidemiological risk of suicide increases, especially in the initial weeks post-discharge (4, 5). A systematic review of post-discharge interventions revealed that improved interprofessional collaboration (IPC) among involved health professionals, such as general practitioners (GPs), psychiatrists, and social services, is needed (6). Similarly, transitions between primary and specialist care, such as referrals from GPs to mental health services, may lead to lost opportunities for early intervention due to delays, unclear responsibilities, or a lack of follow-up (7, 8).

Against this background, well-established IPC has emerged as a promising approach in suicide prevention to ensure that collaboration is appropriately organized, consistently implemented, and able to bridge gaps in care for individuals navigating complex service systems. While evidence suggests that IPC-based models can improve continuity and outcomes in primary care settings, particularly for suicidal ideation and behavior, their effectiveness in reducing suicide deaths remains less well established (9, 10). IPC refers to structured, coordinated collaboration between professionals from different disciplines who jointly contribute to assessment, care planning, and interventions (11). Implemented effectively, IPC facilitates earlier recognition of risk, enhances continuity of care, and delivers broader responses that are more appropriate for individuals experiencing a suicidal crisis (12, 13). Over the past two decades, mental health systems in many countries have moved away from siloed services toward integrated care (14). IPC has therefore shifted from being merely a model to evolving into concrete professional practices across different groups of health professionals. Currently, global bodies such as the WHO include IPC as a key part of patient-centered mental health care (15).

This development can also be observed in Switzerland, where IPC in recent years has become an increasingly important component of (mental) healthcare. The Federal Office of Public Health (FOPH) actively supports IPC in mental health through the Network for Mental Health (NPG, Netzwerk Psychische Gesundheit). This network aims to connect healthcare, social services, education, and other sectors to strengthen mental health promotion and prevention (16). In parallel, the national funding program “Interprofessionality in healthcare 2017–2020” further aimed to improve IPC in the healthcare system and improve efficiency through targeted project funding and policy support.

These approaches to collaboration across health and social care sectors are also relevant for suicide prevention. The Swiss Action Plan for Suicide Prevention (17) embeds IPC as a key element for cross-sectoral early risk detection, timely referral, and continuity of care for individuals with elevated suicide risk. A 2021 midterm evaluation revealed that the action plan has strengthened connections across health and social care sectors, clarified role definitions, and refined referral processes, whereas both this evaluation and more recent analyses also referred to ongoing challenges in achieving consistent regional implementation (18, 19). The most recent engagement plan for 2022–2024 further emphasized the need for sustained cross-sectoral collaboration and concrete mechanisms to anchor IPC more firmly in practice (20). Current policy recommendations further advocate for sustainable interprofessional structures in Swiss suicide prevention (21). Building on these national policy initiatives, several projects have been initiated, with a strong emphasis on improved IPC for suicide prevention. Notable examples include the Attempted Suicide Short Intervention Program (ASSIP) (22) and its adaptations – ASSIP flex, AdoASSIP, and ASSIP Suisse Romande – which incorporate systematic follow-up, interprofessional coordination, and cross-sector communication. The SERO project (*Suizidprävention Einheitlich Regional Organisiert*) combines systematic suicide risk screening with interprofessional training and formalized coordination pathways (23, 24). Common to these initiatives are features of collaborative care, such as shared documentation tools, referral structures and patient transfer mechanisms, outreach strategies, and joint planning across professional boundaries, all aimed at ensuring continuity of care, particularly during high-risk transitions from inpatient to outpatient treatment.

The promise of IPC often contrasts with its implementation in routine practice. Research has identified persistent barriers, including role ambiguity, differing conceptual frameworks, and competing institutional priorities (25). A further challenge frequently noted is the lack of interoperable information systems, which complicates information exchange across professional and institutional boundaries (26, 27). Differences in training, professional cultures, and communication styles may further hinder collaboration even in motivated teams (28), whereas hierarchical dynamics and unclear responsibilities can erode trust and reduce the likelihood of coordinated, timely intervention (29, 30).

Although there is broad agreement that IPC is important in suicide prevention, existing research has provided limited profession-level insight into how IPC is implemented in routine practice and where bottlenecks are perceived. Several quantitative studies have focused on evaluating specific tools or interventions, such as collaborative care models or safety plans (13, 31), while far fewer studies have explored how IPC is structured, sustained, and perceived by healthcare professionals in suicide prevention (10). As a result, it remains insufficiently understood how core professions, such as psychiatry, psychotherapy and nursing, collaborate with each other and with other actors across organizational boundaries.

This study addresses this gap by examining how IPC is organized and delivered in selected Swiss suicide prevention projects from the perspective of participating professionals, with a focus on the structure of collaboration networks, the relative position and importance of different professions within these networks, and the barriers and facilitators influencing IPC. The analysis aims to generate practice-oriented insights to inform strategies for strengthening IPC and improving coordinated care for individuals at risk of suicide.

## 2 Materials and methods

This exploratory qualitative study, conducted among different service providers involved in IPC for suicide prevention, used semi-structured in-depth interviews and analyzed the data using a thematic analysis guided by the principles outlined by Braun and Clarke. In line with the exploratory aim of the study, the analysis was primarily descriptive, focusing on identifying and organizing shared patterns in participants’ accounts rather than developing highly interpretive or theory-driven themes (32). The study is reported in line with Standards for Reporting Qualitative Research (SRQR) and relevant items of the Consolidated Criteria for Reporting Qualitative Research (COREQ) checklist (33, 34).

### 2.1 Eligibility and recruitment

The interviews were conducted as part of an external evaluation of four suicide prevention projects implemented between 2020 and 2024: AdoASSIP (PGV03.083), ASSIP flex (PGV03.062), ASSIP Suisse Romande (PGV03.034), and SERO (PGV03.033). These initiatives not only provided training and courses for professionals involved in the care of individuals at increased risk of suicide but also sought to strengthen IPC to improve the effectiveness of suicide prevention. In particular, ASSIP flex and AdoASSIP emphasized from the outset the importance of establishing effective referral and follow-up practices between hospital and ambulatory care, requiring well-established referral mechanisms and strong IPC to ensure continuity of care.

The professional target groups of the suicide prevention projects included psychiatrists (adults, children, and adolescents), psychologists and psychotherapists, psychiatric nurses, pediatricians and GPs, social workers and social pedagogues, home care providers (Spitex), emergency care professionals working in psychiatric or medical emergency units, and staff of telephone helplines. Although the broader external evaluation also included patients and relatives, the present study analyzes the professional component of the evaluation and therefore focuses exclusively on professional stakeholders. Recruitment followed a purposive strategy to capture a broad range of perspectives on IPC in suicide prevention. At the conclusion of the training and courses offered as part of the suicide prevention projects, professionals were invited to indicate their willingness to participate in an interview. Those who expressed interest were contacted by email, received detailed study information, and returned a signed consent form prior to scheduling a one-on-one Zoom or Microsoft Teams interview. Because participation was based on voluntary self-selection following the training activities, information on non-participation or reasons for declining an interview was not systematically collected. The interviewers had no prior relationship with any of the participants. As participants were recruited from project-related training activities, the final sample composition reflected the distribution of the project training cohorts, resulting in stronger representation from psychologists and psychiatrists compared to other professional groups (see **Table 1**). Accordingly, the analysis does not map the IPC network itself but examines how IPC, including the involvement of other professional actors, was perceived and described by participating mental health professionals.

**Table 1:** Characteristics of the interview participants (N = 15).

Characteristics	Number (n)
Profession	
Psychologist and psychotherapist	9
Psychiatrist and psychotherapist	3
Psychiatric nurse	2
Resident physician	1
Setting^a^	
Outpatient health and social care services	9
Inpatient health care services	7
Type of institution	
Cantonal psychiatric services	8
University hospital-based psychiatric services	6
Ambulatory medical or psychosocial service	1
Project involvement	
AdoASSIP	4
ASSIP flex	3
ASSIP Suisse Romande	3
SERO	5
Region/language	
German-speaking Switzerland	12
French-speaking Switzerland	3
Years of experience	
< 5 years	3
5–10 years	6
11–20 years	4
> 20 years	2
Level of responsibility^b^	
Head of department/senior consultant	1
Consultant/attending psychiatrist	2
Assistant physician/early-career role	1
Licensed psychotherapist/psychologist	9
Psychiatric nurse/specialist nurse	2
Dual role (clinical & research)	2

a One participant worked in both outpatient and day clinic (partial inpatient) settings and was included in both categories.

b Some participants held multiple roles (e.g., therapist and researcher); they were counted under both categories where applicable.

### 2.2 Data collection

Fifteen participants were interviewed. Twelve interviews were conducted in German by the first author, who has a background in suicide prevention and IPC. The remaining three interviews were conducted in French within the framework of the ASSIP Suisse Romande project by the second author, who has expertise in suicide prevention.

A semi-structured interview guide was developed on the basis of the study objectives and project-specific evaluation frameworks. While slightly adapted to the respective project, all the guides addressed IPC and perceived intervention effects (see **Table 2**; full version in **Supplementary File 1**).

**Table 2:** Semi-structured interview topic guide.

#	Topic
1	Understanding and personal definitions of IPC in the context of suicide prevention
2	Experiences with IPC, including key actors and collaboration practices
3	Perceived changes in collaboration due to the intervention(s)
4	Facilitators and barriers to IPC
5	Suggestions for improving IPC in suicide prevention
6	Project-specific reflections (e.g., awareness, visibility, implementation challenges, or unintended effects)

The interviews were conducted between January 2023 and April 2025 and lasted between 34 and 67 minutes (mean = 46 minutes). All interviews were audio-recorded with consent; no one else was present, and no follow-up interviews were conducted. The interviews were transcribed verbatim and pseudonymized. The interviews were conducted via Zoom or Microsoft Teams during participants’ regular working hours. No formal field notes were taken during or immediately after the interviews; instead, analytic memos were generated during the coding and analysis process. Conducting interviews online was chosen to minimize time commitment and financial costs for both the participants and the research team, particularly given the geographic distribution of participants. Given the professional and reflective nature of the topics discussed, in-person interviews were not considered likely to yield substantially different insights or to meaningfully affect data quality. Data collection continued until thematic saturation was judged to have been reached, defined as the point at which no substantively new themes or dimensions relevant to IPC emerged in successive interviews. Saturation was assessed through ongoing analytic memoing during data collection and analysis. While variation in professional background was limited by the composition of the training cohorts, recurring patterns related to roles, coordination mechanisms, and perceived barriers were consistently observed across interviews. The participants were given the opportunity to review their transcripts; however, no one elected to do so. They were not invited to provide feedback on the preliminary findings.

### 2.3 Data analysis

The interview guide was initially developed in German and translated into French for French-speaking participants in the ASSIP Suisse Romande project, using the AI-based tool DeepL Translator (free web version, www.deepl.com; accessed in September 2024). To ensure conceptual accuracy and consistency, the translated guide was reviewed in collaboration with the second author.

The first author transcribed five interviews manually, while the remaining transcripts were produced using the AI-supported tool noScribe. All transcripts, whether manually or automatically generated, were carefully reviewed and corrected by the first author on the basis of the original audio recordings to ensure accuracy and completeness.

To explore reported collaboration patterns across professional groups, a perception-based network visualization was developed. References to IPC were extracted from the interview transcripts and qualitatively categorized based on prominence, contextual relevance, and how collaboration was described by participants. Dyadic connections were coded as strong, moderate, or peripheral, based on the salience and descriptive emphasis of collaboration in participants’ accounts, rather than on numerical thresholds or formal frequency counts. These qualitative classifications informed the visualization: line thickness reflected the interpretive strength of reported collaboration, while node positioning served as visual heuristics to represent the perceived centrality of different professional groups in coordinating care, as described by participants. Centrality is therefore understood as a relational and interpretive concept reflecting participants’ perspectives, not as a formal network metric. The qualitative coding criteria used to classify interprofessional links and the resulting dyadic classifications underlying the network visualization are provided in **Supplementary File 2** (**Supplementary Table 1**, **S2**). The network was visualized using Python’s NetworkX package, with ChatGPT-4o (OpenAI) assisting in the development and refinement of the visualization code. Profession labels were harmonized across transcripts and translated into English for visualization purposes.

In parallel, the data were analyzed using a thematic analysis guided by the principles outlined by Braun and Clarke (32). After in-depth familiarization with the material through repeated readings and analytic notetaking, inductive codes were generated via MAXQDA software (version 24). Codes were developed without a predefined coding frame and remained close to the participants’ own wording. In subsequent phases, related codes were grouped into candidate themes, which were iteratively reviewed and refined to ensure analytic clarity and coherence. In line with the exploratory aim of the study, themes were developed as primarily descriptive analytic categories that organize shared patterns and meanings in participants’ accounts, rather than as highly interpretive or critically reflexive constructions. For example, inductive codes referring to shared clinical focus, continuity across settings, and reduced interface problems were clustered into a broader analytic category capturing IPC as continuity of care. Similarly, codes describing shared responsibility, complementary expertise, and overlapping professional roles were synthesized to reflect IPC as a form of collective accountability across professions. A simplified coding tree and an example illustrating how inductive codes were synthesized into higher-order analytic categories are presented in **Supplementary File 3** (**Supplementary Figure 1**; **Supplementary Table 3**). Both commonalities and divergences across projects and professional roles were examined. The final themes were clearly defined and supported with illustrative quotes to ensure transparency and to reflect participants’ perspectives.

### 2.4 Researcher characteristics and reflexivity statement

The first author was solely responsible for coding and analysis. She has an academic background in public health and health sciences and is currently a PhD candidate in Public Health/Epidemiology, with research experience in IPC and suicide prevention. Her professional background informed the development of the interview guide and the interpretation of findings but was reflexively considered throughout the analytic process. Reflexivity was supported through the systematic use of analytic memos, iterative documentation of coding decisions, and repeated revisiting of the data to check for consistency and alternative interpretations. These measures aimed to enhance transparency, credibility, and rigor (35).

## 3 Results

The findings are presented in three parts. First, the characteristics of the interview participants are described to contextualize the sample. Second, professional roles and their collaborative functions in suicide prevention are examined, along with a network visualization based on participants’ reported interaction patterns. Finally, key themes from the content analysis are presented and illustrated with selected quotes.

### 3.1 Participants

The sample included 15 healthcare professionals working in suicide prevention (see **Table 1**). Participants included psychologists and psychotherapists (n = 9), psychiatrists (n = 3), psychiatric nurses (n = 2), and one resident physician. Participants were employed in outpatient health and social care services (n = 9) as well as inpatient health care services (n = 7). With regard to institutional context, participants worked in cantonal psychiatric services (n = 8), university hospital–based psychiatric services (n = 6), and ambulatory medical or psychosocial services (n = 1). Participants were involved in the suicide prevention projects AdoASSIP (n = 4), ASSIP flex (n = 3), ASSIP Suisse Romande (n = 3), and SERO (n = 5). Most participants were based in German-speaking Switzerland (n = 12), while three worked in French-speaking Switzerland. Participants reported between less than five years and more than 20 years of professional experience and held a range of levels of professional responsibility, from early-career roles to senior positions such as attending psychiatrists and heads of departments. Some participants held dual clinical and research roles. In addition, one participant worked in a day clinic setting, spanning both inpatient and outpatient care and was therefore included in both categories. All of the interviewed participants were actively involved in the care of individuals at risk of suicide through psychotherapy, psychiatric care, crisis services, or specialized suicide prevention programs.

### 3.2 Interprofessional roles and collaboration network as described by interviewed professionals

In the interview data, participants described a broad range of professional groups involved in suicide prevention, each with specific roles and collaborative functions. The classification reflects both participants’ self-rating of their involvement in suicide-related care and the degree of their perceived collaborative functions (see **Table 3**).

**Table 3:** Professional roles involved in suicide prevention and their perceived functions, as described by the interview participants.

Category	Profession/Role	Role in suicide prevention
Mental health professions	Psychiatrists	Provide diagnosis and medical treatment; often responsible for crisis assessment and hospitalization decisions.
	Psychologists and psychotherapists	Offer psychotherapeutic support, psychological testing, and supervision; play a key role in risk assessment, coordination, and ongoing treatment.
	Occupational/movement therapists	Support daily functioning and emotional stabilization; primarily involved in inpatient or rehabilitation settings.
	Complementary medicine practitioners	Provide alternative treatments (e.g., kinesiology); may require additional training in suicide risk recognition.
	Nurses/nursing staff	Deliver hands-on care and monitoring; often first to detect changes in patients’ mental state, assess suicidality in inpatient care and initiate emergency response.
Medical primary care	GPs	Frequently function as the first point of contact; responsible for initial detection, basic intervention, and referral to specialized services.
	Home care providers (e.g. Spitex^a^)	Provide in-home care, often in coordination with primary care teams; often contribute critical observations and crisis management in real-life settings.
Social and legal services	Social workers	Address social, legal, and financial issues; play a key role in connecting individuals to support systems.
	Social pedagogues/Youth care institutions	Assist in supporting families and adolescents; involved in behavioral management and follow-up care.
	Child and Adult Protection Authorities (KESB, *Kindes- und Erwachsenenschutzbehörde*)/Legal guardians	Provide legal oversight and ensure protective measures in cases of elevated suicide risk.
	Probation services/Justice system	Engage in the care of individuals in forensic settings; support reintegration and coordination with clinical services.
Educational system	Teachers/School staff/School social workers	Identify early signs of distress among youth; initiate referrals and collaborate with mental health services.
Public safety and emergency	Police/Emergency services	Respond to acute crises; collaborate with healthcare providers to ensure immediate safety and intervention.
Community and informal care	Residential support staff/Group home caregivers	Provide consistent care and supervision in long-term or residential settings; crucial for daily monitoring.
	Family members/Informal caregivers	Offer emotional support and often observe critical changes in behavior; may serve as contact persons in crisis and frequently seek help themselves.
	Employers	Seldom involved due to confidentiality and stigma; occasionally participate in return-to-work planning.

a Spitex refers to hospital-external home care services in Switzerland (*spitalexterne Pflege*), which may be provided by nonprofit organizations or private providers. Services include nursing care, personal assistance, and support for individuals living at home. No formally designated case manager role was reported; coordination was usually assumed by treating professionals.

In participants’ accounts, psychologists, psychiatrists, and psychiatric nurses emerged as central clinical actors, frequently cited for their role and direct involvement in therapeutic processes, psychiatric assessment, crisis intervention, and coordination of care. In contrast, GPs, teachers, school social workers, and complementary therapists were portrayed as more peripheral as their involvement was typically episodic, context dependent, and focused on initial detection, referral, or follow-up support.

Professions such as social workers, probation officers or police officers, were described as acting at the interface between systems, particularly in situations involving legal protection, community outreach, or acute risk. While not always integrated into formal treatment teams, these actors played a critical role in bridging institutional boundaries. Interviewees did not refer to a formally established “case manager/coordinator” role. Instead, terms such as “key worker” or “primary contact person” (*Bezugsperson*) were used in some instances to denote responsibility for an individual case. However, coordination responsibilities of care processes were generally attributed to psychiatrists, psychotherapists, or psychiatric nurses within their respective treatment settings.

**Figure 1:**
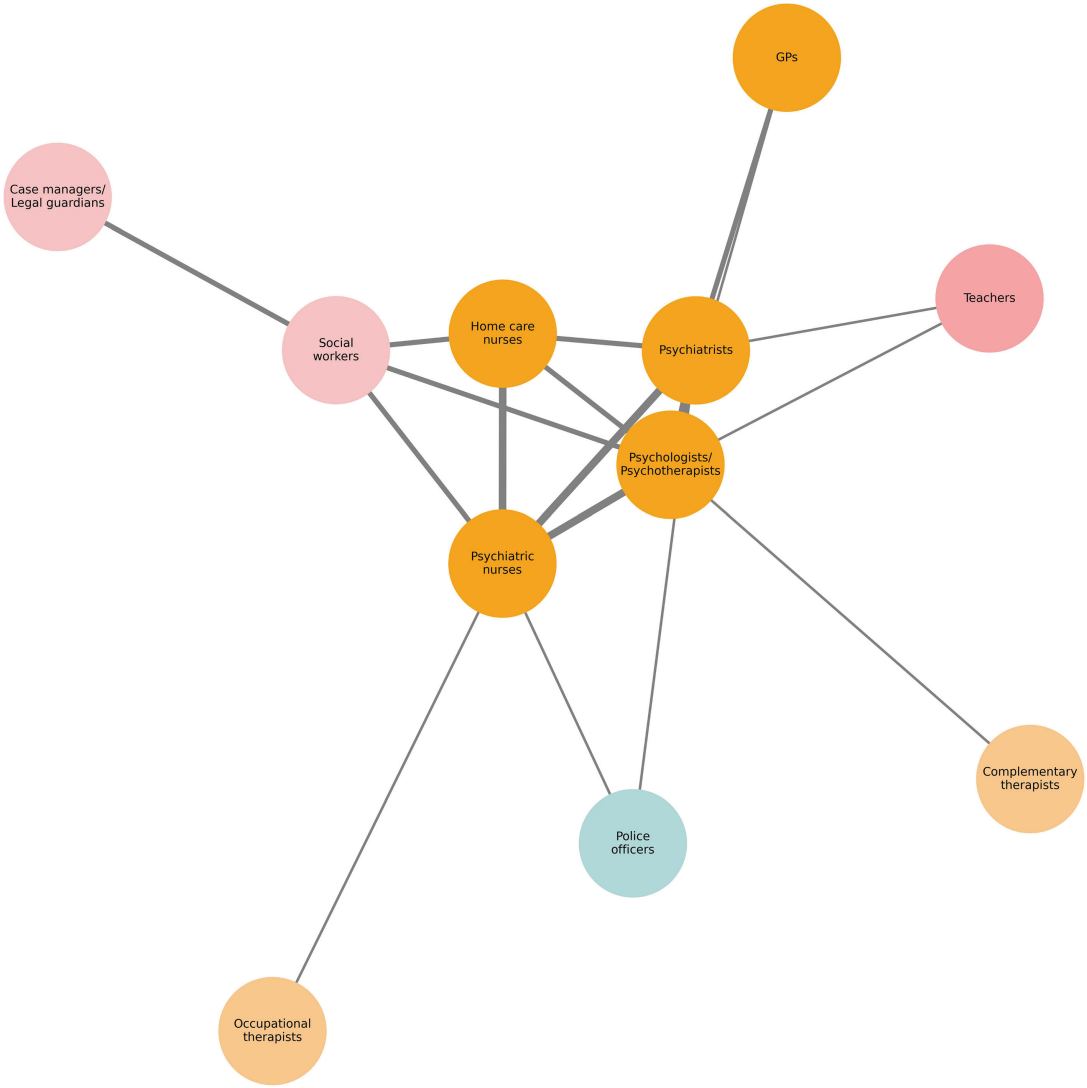
Perceived network of IPC in suicide prevention as described by interview participants. Nodes represent professional groups salient in participants’ accounts, grouped and color-coded by sector. Line thickness indicates the qualitatively coded prominence of collaboration links based on interview material. Node positioning reflects perceived centrality in coordinating care. Professions mentioned less frequently or only in specific local contexts are excluded from the visualization to maintain readability and interpretability but are listed in **Table 3** for completeness. Sector groupings: • Medical professions: psychiatrists, psychologists/psychotherapists, psychiatric nurses, GPs, home care nurses. • Social services: social workers, legal guardians, probation officers, and child protection staff (in the context of adolescents). • Education: teachers (in the context of adolescents). • Community actors: police officers. • Other therapists: occupational and complementary therapists.

Building on the overview in **Table 3**, **Figure 1** visualizes the perceived collaborative relationships reported between professional groups involved in suicide prevention emerging from the interviews. The visualization is based on participants’ descriptions of interaction frequency and intensity (see methods section), with line thickness indicating the perceived strength and frequency of collaboration and node position reflecting the relative centrality of each profession within the perceived interprofessional care system. These patterns reflect participants’ perspectives and should not be interpreted as objective measures of professional engagement.

In participants’ accounts, three professional groups – psychologists/psychotherapists, psychiatrists, and nurses – emerged as the central actors in this network. These professions were not only frequently mentioned in relation to one another but also presented the highest number of connections to other roles. This perceived centrality reflects their sustained involvement in psychotherapeutic treatment, psychiatric assessment, and crisis intervention.

The second tier described by participants included social workers, GPs, and home care providers (including Spitex). These actors were described as maintaining moderate to strong relationships with core professions and were considered essential for care coordination, referrals, and support in non-hospital settings. Although their involvement was described as more episodic, it was nonetheless meaningful and often pivotal in facilitating transitions across services.

Professionals such as legal guardians, probation officers, or staff from child protection services, teachers (in the context of adolescents), occupational therapists, and complementary therapists, were described as context-specific collaborators, typically involved in educational, legal, rehabilitative, or alternative care settings. Police officers were described as playing a bridging role, linking clinical teams with emergency and public safety services during acute crises.

Overall, the network illustrates a layered system of collaboration as reported by interviewees, with varying degrees of involvement across professional roles, with some well-established and some fragmented or underutilized collaborative pathways.

### 3.3 Thematic analysis of IPC in suicide prevention

This section presents the third part of the results, focusing on qualitative findings from the interviews. The themes were developed inductively from the data but are presented here in the order of the interview guide, covering definitions, current practices, perceived facilitators and barriers, and suggestions for improvement, to ensure clarity and coherence in reporting.

#### 3.3.1 Theme 1: understanding and importance of IPC

Across accounts, IPC was described as a fundamental element of suicide prevention, repeatedly mentioning the integration of different perspectives across professional roles. Collaboration is often understood as relationship- and communication-based, rooted in mutual respect and clearly defined responsibilities.

*“It’s the collaboration of different professional groups to bring in different perspectives. [ … ] A social pedagogue, for instance, has a completely different perspective than a psychiatrist, and when we bring that together at eye level, it really strengthens support for the patient.”* (Participant 2, psychiatrist and psychotherapist)

*“Depending on competence, one profession is more involved than another [ … ]. It’s about making the best use of our joint resources for the benefit of the patient.”* (Participant 6, psychologist and psychotherapist)

Some participants expressed concern about overstretching the concept of IPC, particularly in contexts of staff shortages or institutional pressures. In these accounts, IPC was not only framed as collaboration across professions, but also as a site of tension between professional specialization and pressures toward generalization or task redistribution, raising questions about role clarity and responsibility.

*“There’s a risk of lumping everyone together and saying everyone can do everything. That, to me, is not professional collaboration. We each have our training and responsibility. [ … ] These ideas usually arise out of necessity, especially in times of medical staff shortages, but they blur boundaries.”* (Participant 7, resident physician)

The notion of IPC was not limited to intra-institutional teams. Many described it as network-based collaboration extending across sectors and beyond professional boundaries, including close collaboration with family members.

*“For me, it’s about working in a network – with GPs, treating psychiatrists, and also across departments in our own institution. IPC means having those structures that allow us to coordinate across interfaces. We often set up meetings with external caregivers and family to understand the crisis and plan care post-discharge.”* (Participant 10, psychiatric nurse*)*

Collaboration was seen as valuable not only for patients but also for professionals.

*“The fact that we collaborate and come from different backgrounds allows us to really be there for the patient in a meaningful way. [ … ] If professionals already know about* sp*ecific risk situations, they can recognize them faster and protect the person more effectively.”* (Participant 10, psychiatric nurse)

*“For me, it’s a huge relief. [ … ] It’s helpful when another professional joins the process and brings in a new perspective. Especially in situations involving suicidality, responsibility can become a heavy burden – and it matters how that responsibility is shared and distributed across shoulders.”* (Participant 12, psychiatric nurse)

#### 3.3.2 Theme 2: experiences with IPC – key actors and practices

Participants’ accounts converged on a distinction between collaboration with clinical and non-clinical collaboration partners. IPC within mental health care settings – especially among psychiatrists, psychologists, and nurses – is typically seen by participants as smooth and well established. In contrast, collaboration with external actors such as schools, social services, child protection services, or community care presented recurring challenges for interviewees, primarily due to uncertainty and insecurity in dealing with suicidality, particularly regarding responsibility, confidentiality, and the perceived consequences of taking action.

*“With schools, there’s often a lot of fear – fear of saying the wrong thing or of triggering something. [ … ] When a student confides in a teacher, the teacher sometimes believes they cannot tell the parents. But if there’s risk, they must inform them – and that creates uncertainty.”* (Participant 2, psychiatrist and psychotherapist)

*“There is sometimes the expectation that we guarantee nothing will happen. [ … ] We were asked to confirm a suicidal student would be safe going on a school trip. Of course, no one can give such a promise.”* (Participant 9, psychologist and psychotherapist)

Beyond institutional boundaries, the quality of collaboration was described as highly dependent on interpersonal relationships and individual attitudes.

*“It doesn’t really depend on the profession, but more on the working relationship between individuals. [ … ] I think it depends a lot on how well-informed and experienced someone is. With more experience, there is more pragmatism and less fear when talking about suicidality.”* (Participant 6, psychologist and psychotherapist)

#### 3.3.3 Theme 3: structural enablers of and barriers to IPC

The participants pointed to several structures that supported IPC from their perspective, particularly within institutional frameworks. Tools such as the PRISM-S (Pictorial Representation of Illness and Self Measurement–Suicidality), a visual instrument for assessing the degree of suicidality, regular interdisciplinary meetings, and local suicide prevention strategies, were seen as important enablers of shared understanding and coordinated care.

*“We use the PRISM-S to document suicidality risk factors systematically. It helps ensure that everyone [ … ]* sp*eaks the same language. [ … ] It brings clarity and makes task sharing easier not just within the team but also with the affected person.”* (Participant 5, psychologist and psychotherapist)

*“We have suicide prevention meetings every six months at the cantonal level. Different professions come together, and it really helps to build a shared understanding. [ … ] These informal exchanges create a bridge between realities and foster mutual understanding.”* (Participant 13, psychologist and psychotherapist)

However, many noted that these structures were not equally available across all settings. In outpatient care or rural regions, IPC often remains informal and relies on personal initiative.

*“Outside of hospitals, there are often no real structures in place. It’s very much up to the goodwill and network of the individual professional.”* (Participant 8, psychologist and psychotherapist)

Time and financial resources emerged as major barriers, particularly in outpatient services.

*“The time investment is an enormous barrier – and it’s not reimbursed. [ … ] You simply don’t get paid for collaboration. It’s all on top of everything else.”* (Participant 14, psychologist and psychotherapist)

*“Coordination takes time, but no one truly plans for it. [ … ] It’s necessary time, but it’s not formally accounted for.”* (Participant 5, psychologist and psychotherapist)

Structural fragmentation and a lack of shared documentation platforms were not only described as coordination barriers for professionals, but also as an additional burden for patients, who may be required to repeatedly recount sensitive experiences across settings.

*“We don’t have a common platform – everyone uses their own tools. [ … ] That makes communication across institutions difficult.”* (Participant 3, psychologist and psychotherapist)

*“I think it’s not easy for most people to talk about these things. And if the key professionals in the care network already know what is going on and exchange information with each other, that takes a lot of burden off the patient. [ … ] Having to explain oneself again and again would be reduced if professionals communicated better. And if the network is aware of risk factors and difficult situations that are particularly risky for the patient, [ … ] that alone would already improve patient protection.”* (Participant 4, psychologist and psychotherapist)

Some mentioned the absence of political support or systemic incentives.

*“There is no real incentive structure. If someone doesn’t want to collaborate, nothing happens. [ … ] We need tools and guidance that are accessible to everyone.”* (Participant 14, psychologist and psychotherapist)

#### 3.3.4 Theme 4: priorities for strengthening IPC

While framed as recommendations, participants’ accounts reflected shared interpretations of where IPC currently breaks down and what conditions they considered necessary for sustainable collaboration at the institutional, technical, educational, and policy levels. A recurring request was to establish protected time and structures for regular interdisciplinary dialogue.

*“We need structured* sp*aces for interdisciplinary discussion, not just ad hoc solutions when something goes wrong. [ … ] Ideally, this would include diverse professionals, not only psychiatrists. Something like a Balint group could be useful – a protected* sp*ace to reflect on complex cases with GPs, internists, and others.”* (Participant 6, psychologist and psychotherapist)

Institutional support, such as valuing IPC time in schedules, was seen as a decisive factor.

*“In a busy routine, you wonder whether it’s worth attending [such meetings]. However, if the institution sees it as valuable and supports it through working hours, that makes a big difference.”* (Participant 4, psychologist and psychotherapist)

*“Collaboration works best when it’s not just expected, but actually supported – with time, training, and recognition.”* (Participant 13, psychologist and psychotherapist)

Additionally, they called for a shift in how IPC is resourced and valued in everyday practice.

*“IPC is time-consuming – calling schools, coordinating [ … ]. It needs to be recognized and factored into workloads.”* (Participant 5, psychologist and psychotherapist)

The participants also emphasized training needs across sectors, bringing together different professionals, such as teachers, social workers and healthcare staff:

*“There’s still a lot of fear about even addressing the topic of suicidality. [ … ] Teachers, social workers – they often are uncertain what to do.”* (Participant 14, psychologist and psychotherapist)

*“Many still believe talking about it could trigger something, which is not true. [ … ] Short awareness sessions can help professionals feel more secure – even just two hours can make a difference.”* (Participant 15, psychiatric nurse)

A further recurring pattern concerned the redistribution of responsibilities. Suicide risk assessment, for example, was traditionally the exclusive task of physicians, but with tools such as PRISM-S, psychiatric nurses or assigned key workers could now also conduct assessments, while physicians retained ultimate responsibility for decisions such as hospitalization.

*“Before, the risk assessment was entirely up to the doctors. Now, with PRISM-S, the key worker or nurse can do it as well – but the physician still has the final responsibility.”* (Participant 6, psychologist and psychotherapist)

In some interviews, participants referred to the role of key workers who could take on responsibilities extending beyond narrowly defined professional boundaries. This was not a standardized role but rather described as a project-specific function in which a professional was assigned overall responsibility for a patient, depending on experience and availability of expertise.

*“We call them key workers. Each patient is assigned to a professional who takes overall responsibility, regardless of whether the tasks are more social or clinical. The team decides who is best suited based on experience, and additional expertise is brought in when needed.”* (Participant 7, resident physician)

## 4 Discussion

This study combined a perception-based network visualization and a thematic analysis to examine how IPC is organized and experienced in suicide prevention in Switzerland from the perspective of participating mental health professionals. The findings primarily reflect specialist perspectives on IPC, as the sample is weighted toward specialist mental health professions, and challenges specific to primary care, social work, or other low-threshold settings may be underrepresented.

The network-based analysis suggested that, according to the interviewees, IPC for suicide prevention is organized around the core of psychiatrists, psychologists, and psychiatric nurses, consistent with international network studies on the prevention and management of suicidal thoughts and behaviors, which show that specialist mental health professionals are structurally central due to their gatekeeping role in assessing suicide risk and therapeutic decision-making (8, 36). Their close connections in the network reflect shared workplace environments and common professional language – patterns similarly found in child mental health networks (37) as well as among providers who share care of the same patients in mental health services (38). This centrality also reflects the historical development of psychiatry and suicide prevention in Switzerland, where specialist mental health professions traditionally hold a dominant role, whereas in other contexts such as North America or Asia, community actors often play a more prominent role (39).

A second tier of actors comprising social workers, GPs, and home care nurses was described as forming critical bridges at points of transition, such as admission to or discharge from inpatient care. Similar bridging roles for primary care and social work have been documented in suicide prevention networks, particularly during post-discharge follow-up (6, 8). Actors such as teachers, probation officers, and police were described as entering the network episodically, usually in connection with acute events, reintegration challenges, or legal and social support needs, reflecting patterns also observed in community crisis intervention models (40, 41). Interfaces with other systems, such as justice, child protection, or prison services, have been identified in the literature as challenging areas for coordination in suicide prevention, as they involve complex issues of communication and confidentiality between clinicians and non-clinical authorities (42).

The layered structure of professionals involved in suicide prevention suggests, in participants’ accounts, a dependency on a small number of professions that function as central hubs, meaning that they are most frequently connected to others and assume pivotal roles in patient care. While these central professions are often the first point of contact for coordination during acute crises, they were not described as systematically coordinating patient pathways over time. Rather, coordination was seen as occurring situationally within their own setting, without a designated role overseeing processes across sectors. Only in some ambulatory contexts did participants describe the function of a key worker who assumed overall responsibility for a case, but these accounts remained isolated and were not described as systematic or institutionalized. The absence of a clearly established coordination role in this context is noteworthy, given that international evidence highlights care coordination as a central pillar of effective and efficient suicide prevention systems (43–45). While the concentration of responsibilities on central professions may facilitate rapid communication in emergencies, it also risks overloading these professions and leaves collaboration vulnerable to resource constraints or staff turnover, a risk identified in prior evaluations of mental health service networks (46, 47). A further limitation of such concentration is that suicide prevention remains largely confined to psychiatric settings; after discharge, affected individuals often face significant challenges due to gaps in continuity. These findings underscore the need for suicide prevention efforts that extend beyond specialist care into broader life contexts and trajectories (43).

Thematic findings complement the structural perspective by showing how collaboration is experienced within and between these network layers. IPC was widely described by participants as essential for effective suicide prevention. The participants stressed that no single professional group can adequately meet the diverse needs of individuals at risk of suicide. This perception is consistent with international research findings, which emphasize that the complexity of suicide risk requires coordinated, multi-professional measures to address its social, psychological, and medical dimensions (8, 40). IPC in suicide prevention was also frequently described as extending beyond formal professional boundaries to include family members, informal caregivers, or significant others, especially in post-discharge planning. This reflects a broader characteristic of psychiatric care, where social networks often play a central role in supporting continuity and safety. However, participants noted that this involvement is not always systematically implemented, especially during acute crises in hospital settings.

Our findings also suggest that elements of task redistribution are taking place in practice. For example, suicide risk assessment, a task that was traditionally the responsibility of physicians, most often psychiatrists, is now also performed by psychiatric nurses or assigned key workers when structured tools are in use, with physicians retaining final responsibility for clinical decisions. This redistribution of responsibilities aligns with broader evidence on task‐shifting as a strategy to maintain care continuity and IPC (48) and may also foster a more collaborative approach to risk management, emphasizing shared formulation and joint responsibility for patient care and safety (49). Recent studies further underline the value of such collaborative risk formulation, highlighting that multi-perspective assessments involving both clinical and contextual factors can enhance patient safety and treatment engagement (50). At the same time, existing literature indicates that such collaborative arrangements can be challenged by hierarchical tensions and professional boundary negotiations, especially in crisis situations where decision making authority and accountability become salient. Studies from interprofessional healthcare settings show that overlapping responsibilities may trigger role conflict, professional identity threat, and power asymmetries, which can complicate IPC even when task shifting is intended to support collaboration (51–53).

Collaboration was often portrayed by participants as dependent on individuals and shaped by local contexts, rather than anchored in the formal structures of health service delivery. An issue raised in this regard was the lack of shared information systems, such as common patient records, which limits the ability of professionals to coordinate care across settings. The absence of reliable mechanisms for information sharing was seen as a barrier to continuity and efficiency in IPC, echoing international evidence that joint documentation tools are essential ingredients of effective IPC (8, 28).

These challenges were compounded by substantial regional and institutional variability in IPC implementation, reflecting the decentralized structure of the Swiss health system, in which health policy and service organization are largely the responsibility of the cantons, resulting in heterogeneous governance arrangements and care pathways (17, 54). Accordingly, the structural arrangements described here should be interpreted in light of this context and may not be directly transferable to more centralized systems. However, underlying mechanisms identified in this study, such as reliance on personal professional networks, variation in coordination roles, and the fragility of collaboration across transitions, are likely relevant to other fragmented health systems facing similar coordination challenges (27, 55, 56).

The observed variability and fragility of IPC in Swiss suicide prevention is not unique. Similar patterns are reported internationally, where collaboration often functions well in crisis situations but is less embedded in routine care (57). In the present study, IPC was frequently described as reactive, initiated in instances of acute danger or when a clear intervention pathway was triggered, but less consistent during detection, stabilization, rehabilitation, or maintenance phases. International evidence similarly shows that suicide prevention systems concentrate joint efforts on crises, even though sustained aftercare and continuity of care are critical for reducing risk (4, 58). Evidence from post-discharge follow-up further underscores the importance of continuity, with evaluations of aftercare programs and stepped-care models emphasizing the need for formally designated professionals to coordinate patient pathways across settings (5, 6, 59).

Beyond effectiveness and continuity, these patterns also have implications for equity in suicide prevention. Inequities in suicide risk and access to prevention and care are closely linked to social disadvantage, cultural context, and structural barriers within health and social care systems, which disproportionately affect marginalized and underserved populations (60, 61). Fragmented and poorly coordinated services may further exacerbate these inequities by creating additional barriers to access and continuity of care. Strengthened IPC may therefore function as an equity-relevant mechanism by improving coordination across sectors (62), even though equity was not examined directly in this study.

Several facilitating factors of collaboration emerged from the present study with the clarity of role distribution being an important factor. The participants noted that measures that clearly define responsibilities can reduce duplication of work and strengthen trust in joint decisions. This is consistent with previous studies showing that clearly defined roles strengthen collaboration by reducing uncertainty and professional tension (63, 64). In our data, the clarity of responsibilities was seen as particularly relevant in relation to coordinating care pathways across settings and, in some cases, redistributing tasks when staff shortages required flexibility. Mutual trust, often built over time through repeated interactions, was also identified as essential. In some regions, long-standing professional networks enable swift, informal communication that helps resolve crises efficiently, reflecting findings that sustained professional relationships enhance IPC effectiveness in mental health systems (27, 65).

A recurring theme was the role of shared training opportunities and standardized tools in creating a common language for assessing and managing suicidality. Training formats that introduced assessment tools such as the PRISM-S or addressed the clinical handling of suicidal behavior were described as helpful in aligning practices across disciplines, clarifying professional roles, and reducing reluctance in dealing with suicidality. These shared learning environments provided a common reference point that linked different professional logics and institutional contexts, an effect also noted in the literature on interprofessional education (11, 36). Embedding interprofessional training into professional development frameworks was seen as a promising lever for sustaining cooperation structures and role clarity beyond the duration of specific interventions, while also fostering greater confidence among professionals in addressing suicidal issues. An example of such interprofessional training has been established in French-speaking Switzerland, aimed at equipping frontline workers from diverse backgrounds (e.g., doctors, nurses, teachers, psychologists, police officers, social workers) with a shared approach to suicide prevention.

Barriers to IPC were also prominent. A recurrent theme was the reliance on individual initiative and personal networks to maintain collaboration. Owing to a frequently noticed lack of formal structures, IPC often relies on motivated individuals who proactively establish and maintain cross-sector relationships. While such efforts can be very effective in certain contexts, they are inherently fragile: when individuals change positions or retire, collaborative relationships can weaken or collapse. This dependence on individuals has also been observed in other mental health care and primary care systems, where the success of collaboration often depends more on key individuals than on firmly established processes (66, 67).

Resource constraints constituted another key barrier. IPC is time intensive and requires joint meetings, phone calls, documentation, and coordination. Several participants noted that because these collaborative activities are rarely accounted for in workload calculations, interprofessional interaction is often deprioritized under heavy caseloads. Similar dynamics have been reported in interprofessional primary mental health care research (28, 47). This concern is reinforced by the global shortage of mental health professionals, highlighting the need to protect and support IPC within already stretched systems (68). In addition to limited resources, structural fragmentation and incompatible documentation tools often hinder the smooth exchange of information, particularly across institutions using separate systems, and are described as major barriers to effective collaboration (26, 27).

### 4.1 Implications

The findings have several implications for policy and practice. The Swiss Action Plan for Suicide Prevention recognizes the importance of IPC but defers implementation largely to the cantons (17). As reflected in the findings, this appears to contribute to considerable variability, a pattern also observed in other decentralized health systems where regional autonomy results in uneven adoption of collaborative models (27, 57). To move IPC from person-dependent arrangements to embedded, reliable practices, two elements appear crucial. First, formalized structures are needed at the cantonal level, such as protected time and recurring formats for interprofessional dialogue. Cantonal governments, local health services and professional associations are best positioned to promote, establish and maintain such structures. Regular network meetings could help maintain continuity of contact and trust-building beyond the lifespan of individual projects, as demonstrated in evaluations of sustained network interventions in mental health (69). Second, the appointment of care coordinators who assume responsibility for guiding patients in their care seeking pathway could address the current absence of a clearly designated coordination role. Both elements would require shared information systems and financial incentives for collaboration to ensure sustainability. In addition, the establishment of dedicated coordination offices and the integration of flexible funding models across inpatient, outpatient, and home-treatment settings could help close existing gaps at care interfaces. Such measures not only have the potential to improve continuity of care but may also reduce psychopathology and long-term health system costs.

Electronic information-sharing tools also require attention. Incompatible documentation systems hinder collaboration across institutional boundaries, particularly during transitions of care, and limited interoperability has been shown to compromise continuity in other health systems as well (26, 57). Recognizing the coordination time within professional workload planning would additionally legitimize and protect IPC within already stretched systems (28).

Another priority is strengthening ties with non-specialist actors, as many individuals at risk of suicide first present in primary care or educational settings, yet collaboration with these actors is often described as episodic and reactive (2, 41) – an underutilization noted internationally and of concern given that the Swiss Action Plan for Suicide Prevention explicitly emphasizes cross-sectoral synergies and early detection (20). Enhanced training for GPs, teachers, and social service providers (such as mental health first aid programs) could help shift prevention upstream, facilitating earlier detection and intervention. Such measures would also help address the fear and uncertainty reported among these professions when dealing with suicidality, which training interventions have been shown to reduce (70, 71).

### 4.2 Strengths and limitations

A strength of this study lies in the combination of a perception-based network visualization and thematic analysis, which together provide complementary insights into how interprofessional collaboration in suicide prevention is perceived and experienced by participating professionals. This dual approach allowed us to relate professionals’ accounts of collaboration practices to their descriptions of roles, coordination processes, and perceived structural gaps. The interviewers had no prior relationship with the participants, which may have helped minimize bias in the data collection.

Several limitations should be considered when interpreting the findings of this study. First, the sample was weighted toward psychologists and psychiatrists, reflecting the composition of the project-related training cohorts from which participants were recruited. As a result, other professional groups such as primary care providers, social workers, and professionals in low-threshold or community-based settings were underrepresented or described only indirectly through participants’ accounts. Consequently, the findings primarily reflect specialist mental health professionals’ perspectives on IPC and may underrepresent challenges encountered in non-specialist or peripheral settings. Although both inpatient and outpatient sectors were represented, experiences from professionals working predominantly in primary care or other low-threshold services may be insufficiently captured. Second, the study focuses exclusively on professional perspectives. This focus was intentional and aligned with the analytic scope of the external evaluation in which the study is embedded, but it limits insight into how IPC is experienced by patients and relatives. In addition, the network representation reflects reported and perceived collaboration patterns as described by participating professionals rather than objective measures of intersectoral collaboration. Centrality, involvement, and peripheral roles should therefore be interpreted as perspective-based rather than as indicators of actual system-level engagement. Third, although participants were offered the opportunity to review their interview transcripts, none chose to do so, and participants were not invited to provide feedback on preliminary themes or findings. While this approach is consistent with a reflexive analytic framework, the absence of participant validation may limit insight into how closely the interpretations align with participants’ own understandings. Future research should incorporate the perspectives of patients and relatives to better understand how interprofessional processes translate into perceived continuity, accessibility, and quality of care (49, 72). It would also be valuable to examine the perspectives of actors located at the periphery of IPC networks, whose involvement may be episodic but critical, and to explore how IPC is experienced by marginalized and underserved populations, including its potential contribution to more equitable suicide prevention outcomes.

## 5 Conclusion

In this study, we combined a perception-based network visualization with thematic analysis to examine how IPC in suicide prevention is organized and delivered from the perspective of participating mental health professionals in Switzerland. The results revealed a layered network in which, according to participants’ accounts, psychiatrists, psychologists, and psychiatric nurses occupy central positions. GPs and social workers were described as acting as bridging actors, and other actors were involved episodically. While collaboration was widely considered essential, it was perceived as being often fragile and uneven and impeded by the absence of coordination roles, fragmented documentation and resource constraints. However, collaboration was considered to be supported by shared training, role clarity, and trust. Taken together, these findings highlight, from a professional perspective, the dependence of effective suicide prevention on the collaboration of several professions and the need to embed IPC in robust structures. Strengthening care coordination, interoperable information systems, and institutional support emerge as key strategies to ensure continuity and reliability in suicide prevention.

## Data Availability

The datasets presented in this article are not readily available because the respondents did not consent to share their data publicly. Requests to access the datasets should be directed to EH, eva.hollenstein@swisstph.ch
