# Supplementary material for: Professional perspectives on roles and structural gaps in interprofessional collaboration for suicide prevention: a qualitative study
Source: Front Psychiatry. 2026 Mar 9;17:1724853. doi: 10.3389/fpsyt.2026.1724853 (PMC13006919; doi:10.3389/fpsyt.2026.1724853)
Supplement: Supplementary file 1 [file DataSheet1.docx]

**Consolidated Interview Guide: Interprofessional Collaboration in Suicide Prevention**

**Introductory Questions**

1. What is your current profession?
2. Where do you work?
3. How long have you been working in this profession?
4. What is your employment level (percentage)?
5. Please describe your role in the care and treatment of individuals at increased risk of suicide in your organization.

**Interprofessional Collaboration in Suicide Prevention**

1. Are you familiar with the term *interprofessional collaboration*? If yes: what does it mean to you?
2. With which professional groups do you primarily collaborate in caring for individuals at increased risk of suicide?
3. With which professional groups do you have less contact?
4. Does collaboration work better with some professional groups than with others? If so: which ones, and why?
5. Do different perspectives or approaches among professionals complicate collaboration?
6. What possibilities exist for coordinating between professions in suicide prevention?
7. In your opinion, how important is interprofessional collaboration in suicide prevention and care for individuals at risk? Why?
8. What do you see as the main benefits of close collaboration for these individuals?

**Project-Specific Section (AdoASSIP / ASSIP flex / ASSIP Romande / SERO)**

1. How did you first learn about the project [insert relevant project name]?
2. Since the project started, has interprofessional collaboration changed in your region? If yes: in what way?
3. What do you consider the most positive effect of the project in terms of interprofessional collaboration?
4. What do you consider the most positive effect of the project for patients?
5. What challenges did the project face in terms of interprofessional collaboration?

**Project-Specific Intervention (if applicable)**

1. Has using the [insert relevant project name] intervention facilitated collaboration within your team? If yes: how?
2. Has it facilitated collaboration outside your team (e.g., between inpatient and outpatient settings)? If yes: how?
3. How has the use of the intervention influenced your personal clinical work (positively/negatively)?
4. (SERO only) How was suicidality assessed before using PRISM-S? Was a specific method applied?

**Recruitment (AdoASSIP only)**

1. Are you satisfied with the number of patients recruited? Did you have specific goals, and were they met?
2. How does recruitment currently take place at your institution?
3. Which recruitment steps are most resource-intensive, and why?
4. Which aspects of recruitment could be improved, and how?
5. What do you see as the main barriers to recruitment (systemic, structural, or personnel-related)?
6. What are essential success factors for recruitment?

**Sustainability & System-Level Change**

1. What needs to be in place to sustain this project after it ends?
2. Have you already made plans to continue its activities? (e.g., integration into routine care, political support, funding, partnerships)
3. What are the greatest challenges in this regard?
4. Where do you already see good support or few obstacles?

**Improvement & Recommendations**

1. What initiatives or measures do you think are needed to improve interprofessional collaboration in suicide prevention?
2. What could be done at the institutional or structural level to support better collaboration? What incentives might help?
3. What factors at the policy or system level should be considered to facilitate interprofessional collaboration?

**Closing**

1. Is there anything else you would like to add on the topic?
