# Supplementary material for: Professional perspectives on roles and structural gaps in interprofessional collaboration for suicide prevention: a qualitative study
Source: Front Psychiatry. 2026 Mar 9;17:1724853. doi: 10.3389/fpsyt.2026.1724853 (PMC13006919; doi:10.3389/fpsyt.2026.1724853)
Supplement: Supplementary file 2 [file DataSheet2.docx]

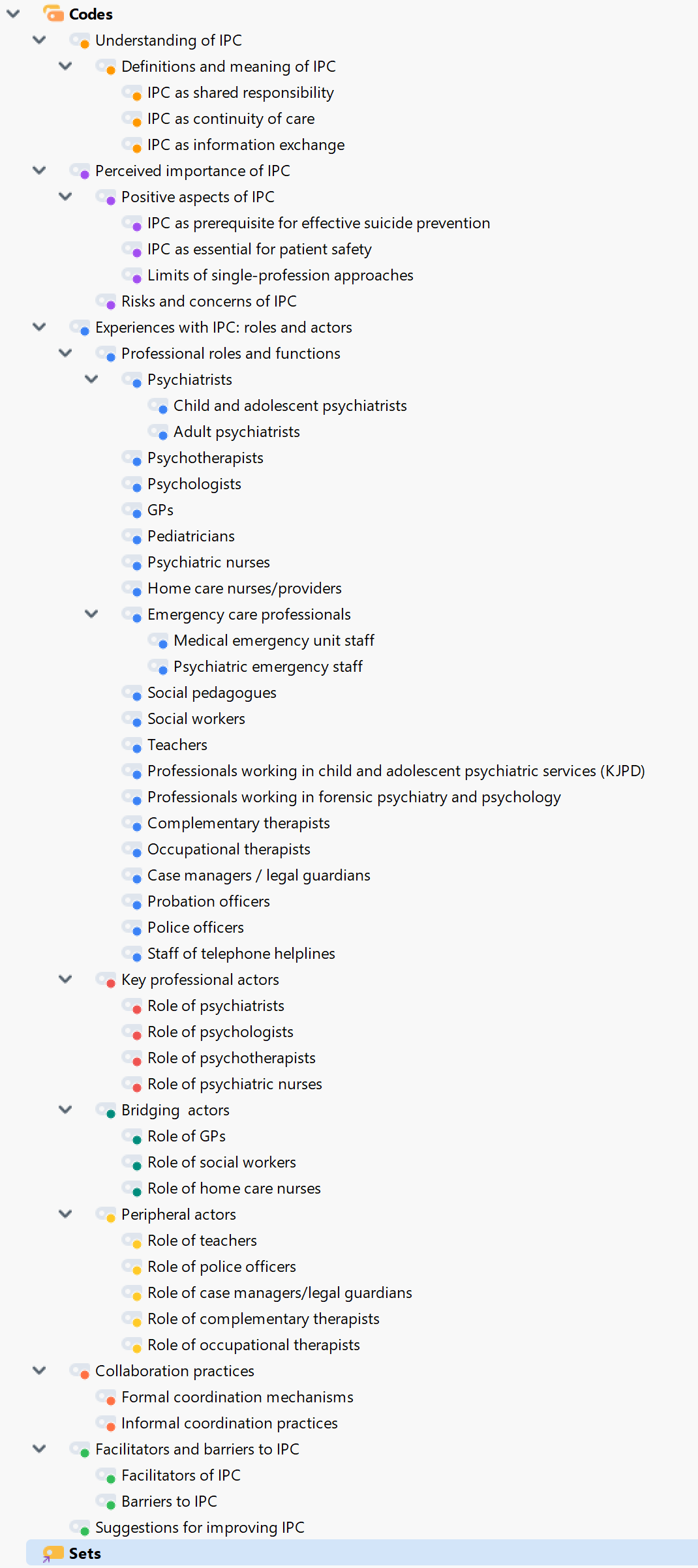


**Supplementary Figure S1.** Simplified coding tree used in the thematic analysis.
The figure presents the hierarchical organization of inductively developed codes, subthemes, and main analytic categories used to analyze participants’ accounts of IPC in suicide prevention. The coding tree is provided for transparency and illustrates how codes were organized into higher-order categories; it does not imply code frequency or relative importance.

**Supplementary table S3.** Example of analytic progressions for main theme 1“Understanding and importance of IPC”

| **Subthemes** | **Codes** | **Subcodes** | **Illustrative participant quote (abridged)** |
| --- | --- | --- | --- |
| Understanding of IPC | IPC as continuity of care | shared clinical focus; continuity of care; reduced interface problems; care optimization | *“I would define it as collaboration with different disciplines or with people from other professional groups who have different professional backgrounds but are working on a shared issue, such as suicide prevention. The aim is to ensure and safeguard care, and ideally to optimize it, for example by reducing interface problems through smooth collaboration.”* (Participant 1, psychologist and psychotherapist) |
|  |  | continuity of care; continuity across settings; care pathway orientation; coordination across interfaces; network-based collaboration | *“In my opinion it’s about working in a network – with GPs, treating psychiatrists, and also across departments in our own institution. IPC means having those structures that allow us to coordinate across interfaces. We often set up meetings with external caregivers and family to understand the crisis and plan care post-discharge.”* (Participant 10, psychiatric nurse) |
|  | IPC as shared responsibility | shared responsibility; task distribution; complementary expertise; collective accountability | *“In my experience, this involves different professional groups who may have different educational backgrounds but care for the same clients and therefore share responsibility. Depending on competence, one profession is more involved than another [...]. It's about making the best use of our joint resources for the benefit of the patient.”* (Participant 6, psychologist and psychotherapist) |
|  |  | complementary expertise; shared responsibility; non-hierarchical collaboration; multi-perspective care | *“For me, it’s the collaboration of different professional groups to bring in different perspectives. [...] A social pedagogue, for instance, has a completely different perspective than a psychiatrist, and when we bring that together at eye level, it really strengthens support for the patient.”* (Participant 2, psychiatrist and psychotherapist) |
|  |  | shared responsibility; collective accountability; overlapping expertise; role complementarity | *Interprofessional work means that although we all have different educational pathways and experiences and each brings different knowledge, responsibility lies with everyone. I see it as overlapping circles of knowledge, with certain areas intersecting across professions, for example between nursing and psychology, alongside profession-specific knowledge that each discipline contributes.”* (Participant 7, resident physician) |
|  | IPC as information exchange | information sharing; interdisciplinary communication; shared clinical focus | *“I am more familiar with the term interdisciplinary collaboration, but I think it is often used synonymously. For me, it primarily refers to the exchange of information and collaboration with professionals from different disciplines, such as physicians, psychologists, or social workers, who are working on the same issue or toward the same goal.”* (Participant 4, psychologist and psychotherapist) |
|  |  | information sharing; professional networking; coordinated care planning; continuity of care | *“I understand it as networking and improving the flow of communication among all professionals involved in the same case. This can involve internal collaboration within a team as well as collaboration with colleagues from outside the organization. In our setting, interprofessional collaboration mainly means that everyone involved coordinates closely to ensure that the patient is received in the best possible way and cared for in the most appropriate manner.”* (Participant 12, psychiatric nurse) |
