# Supplementary material for: Professional perspectives on roles and structural gaps in interprofessional collaboration for suicide prevention: a qualitative study
Source: Front Psychiatry. 2026 Mar 9;17:1724853. doi: 10.3389/fpsyt.2026.1724853 (PMC13006919; doi:10.3389/fpsyt.2026.1724853)
Supplement: Supplementary file 3 [file Table1.docx]

**Table S1.** Interpretive coding criteria used for network visualization

| **Classification of interprofessional link** | **Interpretive criteria based on interview material** | **Quantitative threshold** |
| --- | --- | --- |
| Strong | Collaboration described as central to care processes and routinely enacted, often emphasized as essential for coordination or continuity of care across multiple interviews | Not applicable; classification based on qualitative prominence and contextual emphasis in participants’ accounts. |
| Moderate | Collaboration described as regular but context-dependent, for example activated at specific points such as crises, transitions of care, or case planning, without being embedded in daily workflows. | Not applicable; classification based on qualitative prominence and contextual emphasis in participants’ accounts. |
| Peripheral | Collaboration described as infrequent, episodic, or limited to isolated situations (e.g., emergencies or specific referrals), and not characterized as a core element of routine care. | Not applicable; classification based on qualitative prominence and contextual emphasis in participants’ accounts. |

**Table S2.** Qualitative coding of interprofessional dyads underlying Figure 1

| **Profession A** | **Profession B** | **Classification of interprofessional link** |
| --- | --- | --- |
| Psychiatrists | Psychologists/Psychotherapists | Strong |
| Psychiatrists | Psychiatric nurses | Strong |
| Psychologists/Psychotherapists | Psychiatric nurses | Strong |
| Psychiatric nurses | Home care nurses | Strong |
| Psychologists/Psychotherapists | Social workers | Moderate |
| Psychiatric nurses | Social workers | Moderate |
| Psychiatrists | GPs | Moderate |
| Social workers | Home care nurses | Moderate |
| Psychologists/Psychotherapists | Home care nurses | Moderate |
| Case managers/Legal guardians | Social workers | Moderate |
| Psychiatrists | Home care nurses | Moderate |
| Psychologists/Psychotherapists | GPs | Peripheral |
| Teachers | Psychologists/Psychotherapists | Peripheral |
| Teachers | Psychiatrists | Peripheral |
| Police officers | Psychiatrists | Peripheral |
| Police officers | Psychiatric nurses | Peripheral |
| Occupational therapists | Psychiatric nurses | Peripheral |
| Complementary therapists | Psychologists/Psychotherapists | Peripheral |

Note: Classifications reflect the qualitative coding used to generate the network visualization (Strong, Moderate, Peripheral) and are based on how interprofessional collaboration was described in participants’ accounts. The table documents the analytic input underlying Figure 1.
